# P-957. Incorporating Negotiation Strategies to Enhance Antimicrobial Stewardship Using Artificial Intelligence (INSEAMS-AI)

**DOI:** 10.1093/ofid/ofaf695.1159

**Published:** 2026-01-11

**Authors:** Brandon J Webb, Whitney Buckel, Darrell Childress, Jared Olson, Meagan N Greckel, Rilee Christensen, John J Veillette, Sara Palmquist, Souha Haydoura, Vincent Anella, Rachel Foster, Payal K Patel

**Affiliations:** Intermountain Health, Murray, UT; Intermountain Health, Murray, UT; Intermountain Health, Murray, UT; Intermountain Health, Murray, UT; Intermountain Health, Murray, UT; Intermountain Health, Murray, UT; Intermountain Healthcare, Murray, UT; Intermountain Health, Murray, UT; Intermountain Health, Murray, UT; Intermountain Health, Murray, UT; Intermountain Medical Center, Draper, UT; Intermountain Healthcare, Murray, UT

## Abstract

**Background:**

Effective communication is critical to successful antimicrobial stewardship (AS). Negotiation strategy has been extensively used in other disciplines to enhance communication. We aimed to evaluate whether incorporating negotiation strategies into AS practice would improve AS-prescriber interactions. Since most AS providers do not have access to negotiation training, we also hypothesized that an artificial intelligence (AI) large language model could generate useful examples of AS-tailored negotiation techniques when provided a standardized prompt and a description of a real-life AS scenario.

**Table 1.**
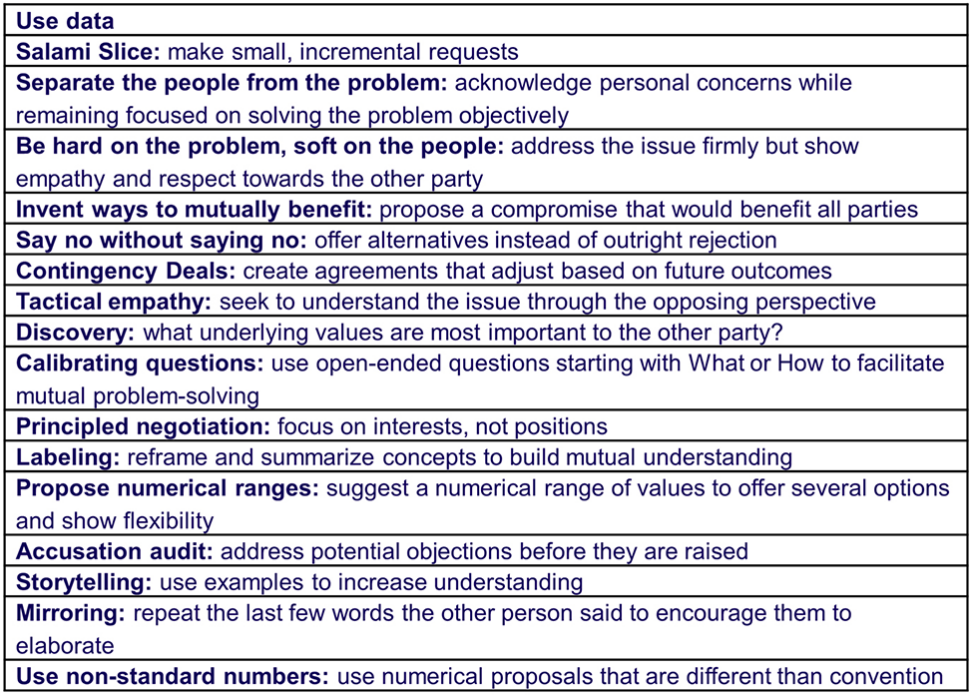
Negotiation Strategies with Applicability to Antimicrobial Stewardship

**Figure 1.**
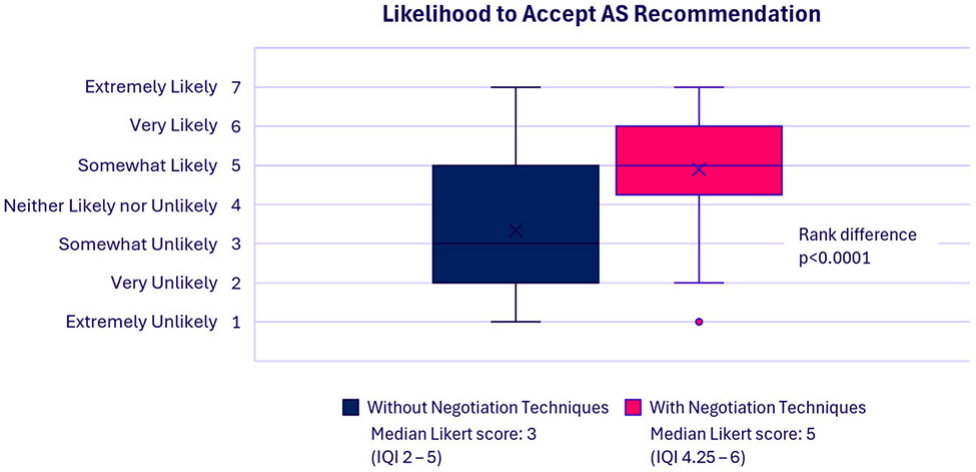
Likelihood to Accept Antimicrobial Stewardship Recommendations

**Methods:**

We identified negotiation strategies (Table 1) most likely to be applicable to AS using a cross-disciplinary literature search. Using generic AS scenarios, we iteratively developed an AI prompt that consistently elicited accurate examples of the negotiation techniques. We assembled a dataset of 18 real-life AS-prescriber interaction scenarios using an anonymized survey. We then used the prompt to generate AI-recommended negotiation techniques for each of the 18 real-life scenarios. A diverse group of ten AS investigators assessed the most likely outcome of each scenario using 7-point Likert-scale questions, first without negotiation techniques, and then again, assuming that the examples of negotiation techniques generated by AI had been available to the AS provider in each interaction. The rank differences test was used to compare pairwise responses.

**Figure 2.**
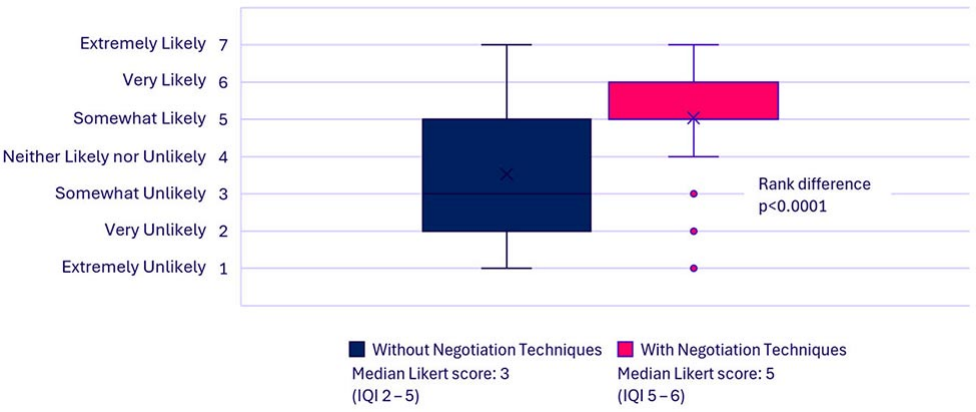
Likelihood of Mutually Agreeable Resolution of the Antimicrobial Stewardship-Prescriber Interaction

**Results:**

AS evaluators assessed that there was a significantly higher likelihood that 1) the AS recommendations would have been accepted (Figure 1), p< 0.0001, and 2) that there would have been a mutually agreeable resolution to the interaction (Fig. 2), p< 0.0001, had AI-suggested negotiation techniques been available for AS providers to use.

**Conclusion:**

Incorporating negotiation strategies into AS communications with prescribers has potential to improve interactions and increase acceptance of recommendations. Leveraging AI as a “real time negotiation coach” may make these techniques more broadly accessible to AS providers. Future opportunities include prospective implementation studies and negotiation training for AS practitioners

**Disclosures:**

Payal K. Patel, MD, MPH, FIDSA, Cormedix: Advisor/Consultant

